# Physical Exercise and Psychophysical Learnings on Basic Strength Development

**DOI:** 10.3390/mps8020040

**Published:** 2025-04-10

**Authors:** Gaetano Raiola, Giovanni Esposito, Sara Aliberti, Francesca D’Elia, Tiziana D’Isanto

**Affiliations:** 1 Research Centre of Physical Education and Exercise, Pegaso University, 80143 Napoli, Italy; gaetano.raiola@unipegaso.it (G.R.); gioesposito@unisa.it (G.E.); tizidisanto@gmail.com (T.D.); 2 Department of Human, Philosophical and Education Sciences, University of Salerno, 84084 Fisciano, Italy; fdelia@unisa.it

**Keywords:** perception, opinions, awareness, hand grip, isometric mid-thigh pull

## Abstract

Strength development through physical exercise enhances neuromodulator production, neural connectivity, and motor unit efficiency. Beyond physical benefits, understanding individuals’ perceptions, opinions, and knowledge can optimize engagement in exercise. However, existing literature lacks studies examining these factors alongside strength development. This study aimed to investigate whether the effectiveness of strength training protocols is associated with individuals’ perceptions, opinions, and knowledge, thereby establishing a link between performance enhancement and awareness of the physiological demands of exercise. The findings seek to highlight the educational potential of physical exercise in promoting psychophysical well-being. A total of 24 participants (14 males, 10 females), aged 35–55 years with varying occupational backgrounds and sedentary levels, were recruited. A strength development protocol was administered, and the participants completed perception-based questionnaires at three time points. Statistical analyses, including repeated-measures ANOVA, Friedman’s test, and post hoc comparisons, were conducted. Significant strength improvements were observed, specifically in the Hand Grip Test (*p* < 0.01). An increase, but non-significant, emerged in the Isometric Mid-Thigh Pull from 1850 ± 210 N to 2270 ± 190 N. The participants also reported a 35% increase in motivation to engage in exercise and a 42% reduction in sedentary behavior. Additionally, 78% of the participants demonstrated greater awareness of exercise benefits, correlating positively with physical improvements. The findings indicate that strength development is associated with increased awareness of the benefits of physical exercise, supporting its use as an educational tool to enhance engagement and adherence to exercise protocols.

## 1. Introduction

Regular physical exercise offers numerous benefits, including slowing the aging process and promoting overall well-being. Extensive scientific research confirms the positive impact of exercise on both physical and mental health [1,2,3,4,5,6]. Despite its proven advantages, physical inactivity remains a major global health concern, contributing to chronic diseases, reduced quality of life, and increased healthcare costs. Physical exercise and physical activity are often used interchangeably. However, physical activity encompasses all bodily movements that require energy expenditure, including daily tasks and recreational activities [7]. Exercise, on the other hand, is a structured, repetitive, and goal-oriented subset of physical activity specifically designed to enhance physical fitness [8]. Both play a crucial role in overall health, but exercise provides targeted benefits that contribute to long-term physiological and cognitive resilience. Recognizing the importance of maintaining an active lifestyle, the World Health Organization (WHO) has developed specific guidelines for different age groups. For children and adolescents (5–17 years), at least 60 min of moderate-to-vigorous physical activity per day is recommended. Adults (18–64 years) should engage in either 300 min of moderate activity or 150 min of vigorous activity per week, alongside at least two days of resistance training [7]. Given the constraints of work and daily responsibilities, it is essential that exercise routines are adaptable to individual lifestyles [9]. For older adults (65+), aerobic exercise, muscle strengthening, and movement-control activities are encouraged to reduce the risk of falls and maintain mobility [10]. Beyond its physiological benefits, exercise plays a critical role in lifelong learning by preserving both physical and cognitive function while mitigating natural age-related decline. To maximize its effectiveness, traditional instructor-led training models must evolve into dynamic, participant-centered approaches that encourage individual engagement and adaptability [11]. Higher education institutions have recognized this need and are integrating advanced training methodologies to develop strength in various forms: maximal, explosive, and endurance-based [12,13]. Strength training not only enhances the musculoskeletal, cardiovascular, and respiratory systems but also promotes neuroplasticity by stimulating neurotransmitter production and improving motor unit connectivity [14,15]. From an educational perspective, strict adherence to predefined exercise protocols may be insufficient for optimizing results [16,17]. Instead, an ecological approach, where individuals actively participate in shaping their training experiences based on their perceptions, opinions, and knowledge, may yield greater long-term benefits. This participatory model fosters a deeper understanding of exercise’s physiological effects, promoting self-awareness and sustainable engagement in physical activity.

This study investigates the relationship between traditional strength development protocols and individuals’ perceptions, opinions, and knowledge of physical exertion. Specifically, it examines whether increased awareness of energy expenditure and exercise intensity contributes to improved performance and long-term psychophysical well-being. Identifying these connections will provide valuable insights into the role of exercise in education, emphasizing the importance of conscious engagement in physical activity for lifelong health and fitness.

## 2. Materials and Methods

### 2.1. Study Participants

Given the purpose and design of this study, a convenience sample of 24 individuals (14 males and 10 females) aged between 35 and 55 was used. This range was motivated by the need to study an age group characterized by significant physiological changes, including a progressive reduction in muscle mass and strength, which can be mitigated through strength training. The participants were drawn from various occupational sectors, including office work, trade, industry, and services, with job roles ranging from administrative and managerial positions to manual labor. This occupational diversity reflects varying levels of sedentary work, allowing for an exploration of how different work habits and lifestyles influence physical activity levels and overall psychophysical well-being. The participants were selected through a simple randomization process from a pool of volunteers who met the inclusion criteria. The inclusion criteria required the participants to: (1) be between 35 and 55 years of age, (2) have no serious chronic conditions or physical disabilities that could interfere with physical activity, (3) be willing to participate in a structured exercise program, and (4) provide informed consent and adhere to the research protocol guidelines.

### 2.2. Study Design

The study design is exploratory and observational and, because it aimed to evaluate the effectiveness of a strength development protocol and the relationship between physical improvements and the participants’ awareness of the benefits of exercise, is wholly original. The participants underwent two physical assessments at the beginning, mid-point (in itinere), and conclusion of this study. To evaluate maximum isometric lower limb strength, the Isometric Mid-Thigh Pull (IMTP) test—a scientifically validated method—was employed [18]. Upper-limb strength was assessed using the Hand Grip Test (HGT), though it has not yet been formally validated [19,20]. The IMTP test utilized a squat rack anchored to the floor, a force platform (Kinvent K-Deltas), and specialized software for data collection and analysis. The participants stood on the force platform with a knee angle of 140° and a hip angle of 120°, gripping a fixed iron bar. They performed a five-second isometric pull, exerting maximal force in the shortest possible time. For the HGT, the participants were seated at a 90° femur-to-torso angle. Their grip strength was measured using a Kinvent K-Grip dynamometer, placed on the knee for support, with the elbow flexed at 90°. Each participant applied maximum effort for five seconds. Between the initial and final assessments, the participants engaged in an eight-week strength development training protocol, consisting of three weekly sessions.

### 2.3. Procedure

The training was conducted in a physiotherapy center gym and was structured into two distinct phases. The first phase, termed the accumulation phase, emphasized high-volume training to develop proper technique and foundational strength. The second phase, known as the intensification phase, involved a reduction in training volume while progressively increasing intensity to optimize strength development. Each session commenced with a 15-min warm-up, incorporating mobility exercises targeting the hip rotators, pelvis, and spine, along with glute activation drills and quadrupedal mobility movements. Dynamic exercises reinforcing key movement patterns, such as squats, hinges, and split squats, were also included. Following the warm-up, the participants engaged in a structured strength training regimen.

The training program consisted of various strength-building blocks. The Light Implement Power Block introduced explosive, low-impact movements, including Non-Counter Movement Box Jumps and Kneeling Chest Passes with a 3 kg medicine ball. The Strength Blocks incorporated exercises such as Goblet squats, 2DB Floor Presses, and Elevated Front Planks, which progressed in intensity over time. Additionally, Single Staggered-Foot Deadlifts and Kneeling Horizontal Pull-Ups were included to enhance both lower and upper body strength. As training advanced, progressive overload was applied, increasing the load of certain exercises by 2.5 kg per week and gradually extending core exercise duration. The intensification phase maintained the same warm-up routine but introduced more demanding variations, such as Counter Movement Jumps and Hip-Hinge Chest Passes. The resistance training intensity was increased through heavier loads and modifications to plank exercises, including knee-off-the-ground variations. At the conclusion of the program, the participants completed a questionnaire assessing their perceptions and opinions regarding the benefits of strength training. The subjective evaluations were subsequently compared with objective strength performance data to identify potential correlations between physical improvements and individual experiences. Table 1 and Table 2 present the specific training protocols followed by the sample.

### 2.4. Survey

At the beginning, during, and at the end of this study, a specially designed questionnaire (Table 3) was administered to assess the participants’ perceptions and opinions on how strength development impacted their daily lives. The questions were adapted at different times in the protocol to reflect the progression of the participants’ experience, but the items measure the same construct, allowing comparison of scores over time (awareness of muscle strength, protocol affecting your daily life or performance, and motivation to perform the protocol). Each question provided five response options (1 = not at all, 2 = a little, 3 = average, 4 = very important, and 5 = absolutely) allowing the participants to select the one that best reflected their experience.

### 2.5. Statistical Analysis

The data are presented descriptively with mean (M) and standard deviation (SD) values. The normality of the data distribution was tested using the Shapiro–Wilk test. Since there were no separate groups, the analysis focused on repeated measures within the same participants. The Friedman test was used to assess differences in the survey scores across the three time points (pre, mid, and post) within the same sample. To further explore specific differences between the time points, post hoc analyses were conducted. The Bonferroni post hoc test was applied to the sit-and-reach data, while the Wilcoxon signed-rank test was used for pairwise comparisons of the survey data. These tests allowed for the identification of significant differences between the pre-mid, pre-post, and mid-post scores. To validate the survey, its internal consistency was assessed using Cronbach’s alpha and associated 95% confidence intervals (CIs). A Cronbach’s of 1 indicated perfect reliability, with a cutoff of 0.7 indicating an acceptable internal consistency. The statistical significance was set at *p* ≤ 0.05. The data processing and analysis were performed using the Statistical Package for the Social Sciences (IBM SPSS Statistics for Windows, version 25.0, IBM, SPSS Inc., Armonk, NY, USA).

## 3. Results

Table 4 presents the results of the HGT measured at three time points: pre-test, mid-program, and post-test. The data reveal a consistent upward trend in grip strength for both the dominant (DX) and non-dominant (SX) hands throughout the training program. The mean strength values for the dominant hand increased from 31.75 ± 8.51 kg at pre to 34.02 ± 8.31 kg at mid, reaching 37.40 ± 8.84 kg at post. Similarly, the non-dominant hand showed significant improvements, rising from 31.05 ± 8.26 kg at pre to 33.83 ± 8.14 kg at mid, and further increasing to 36.97 ± 8.10 kg at post.

The results of the ANOVA for the HGT revealed a non-significant difference in the dominant (DX) hand scores over time (F = 2.65; *p* = 0.07) and the non-dominant (SX) hand scores (F = 3.16; *p* = 0.04). For the non-dominant hand, post hoc analysis revealed significant differences between the initial and intermediate scores (*p* = 0.00) and between the initial and final scores (*p* = 0.00). However, no significant difference was observed between the intermediate and final scores (*p* = 0.06). This suggests that grip strength improvements are statistically significant over time for both hands, with the most pronounced changes occurring between the initial and intermediate phases, as well as the initial and final phases. A detailed description is shown in Table 5.

The results of the IMTP test (Table 6) indicate a progressive increase in mean force output across the three time points. Athletes showed an improvement from 97.69 ± 20.04 kg at the pre to 105.45 ± 20.57 kg at the mid, reaching 108.06 ± 20.26 kg at the post. These findings suggest a continuous enhancement in maximal isometric strength throughout the training program.

The ANOVA results for the IMTP revealed no statistically significant overall effect over time (F = 1.70; *p* = 0.19). This means that the observed changes in IMTP values could be due to natural variability in the data and not necessarily the effect of training.

The internal consistency of the questionnaire was good (Cronbach’s α coefficient [95% CI]: 0.87 [0.81–0.89]; *p* < 0.00). The results, shown in Table 7, indicate an increase in mean perception scores over time. In the pre-test phase, the mean scores were relatively low (Q1: M = 2.41; Q2: M = 2.50; Q3: M = 2.58), while in the mid-test phase, a significant increase was observed (Q1: M = 3.37; Q2: M = 3.41; Q3: M = 3.45), which further increased post (Q1: M = 4.33; Q2: M = 4.41; Q3: M = 4.62). This suggests a perceived improvement over time. The observed change in scores is accompanied by relatively stable standard deviations, indicating that the increase in perception was not random or limited to a few individuals, but rather a consistent change across the participants.

The results of the Friedman test (Table 8) reveal a statistically significant difference in the perceptions of athletes over time (χ^2^F = 8.71; *p* = 0.01). The post hoc analysis, conducted using the Wilcoxon test, indicates a significant difference between the initial and intermediate scores (*p* = 0.00), the initial and final scores (*p* = 0.00), and the intermediate and final scores (*p* = 0.01), suggesting a perceived change over time. The effect size for both pre vs. post and mid vs. post comparisons was moderate.

## 4. Discussion

The study results highlight key insights into the effectiveness of the strength development protocol and its impact on the participants’ perceptions, opinions, and knowledge. Physical exercise is widely recognized as one of the most effective strategies for combating aging and promoting health across all age groups [21]. Maintaining an active lifestyle, even as age advances, can help individuals sustain a high quality of life and prolong independence [22,23]. Strength training, in its various forms, is a fundamental component of exercise programs aimed at counteracting the effects of aging [24]. This study aimed to evaluate whether a structured strength training program could enhance physical performance and to explore whether improved performance correlated with greater awareness of the benefits of exercise. The findings can be analyzed through two primary dimensions: strength development and awareness of physical exercise. The HGT results demonstrated a significant increase in grip strength only for non-dominant (SX) hands between the initial, mid-program, and final assessments. For the dominant hand, the mean force levels improved slightly from 31.75 kg at pre to 34.01 kg at mid and further to 37.39 kg at post. Although ANOVA indicated a positive but non-significant trend in dominant hand strength, the mean values revealed an increase in strength across all assessment points. For the non-dominant hand, the mean force values increased from 31.05 kg at pre to 33.83 kg at mid and 36.97 kg at post, with ANOVA confirming statistical significance, further supported by post hoc comparisons. These findings suggest that the strength development protocol effectively improved isometric grip strength, emphasizing the role of progressive training in neuromuscular adaptation.

Similarly, the IMTP results reinforced the effectiveness of the training program. The mean force levels increased from 97.69 kg at pre to 105.46 kg at mid and 108.07 kg at post. Although the overall ANOVA did not reach statistical significance, the mean values indicated an increase in strength between pre and mid, pre and post, and to a lesser extent, between mid and post. These results suggest that despite individual variability, the training program led to overall improvements in isometric strength. Analysis of the questionnaire data, assessed using a Likert scale, revealed significant changes in the participants’ perceptions and awareness across the three assessment points. The Wilcoxon test identified significant improvements in all the questionnaire items, reflecting an increased understanding of the role of physical exercise in psychophysical well-being. The participants reported greater awareness of exercise as a tool for enhancing quality of life and overall health, increased self-efficacy in performing physical activities, and a more positive attitude toward structured exercise. This suggests a strong relationship between improved physical performance and heightened awareness of exercise benefits. Beyond increasing muscle strength, the training protocol fostered a deeper understanding of the importance of physical activity for overall well-being. This dual impact is particularly relevant in educational settings, where structured exercise programs can be leveraged to teach resilience, engagement, and the long-term value of an active lifestyle. Improved strength levels also contributed to greater knowledge about the role of strength training in slowing the aging process. This finding aligns with previous research indicating that maintaining adequate strength in both upper and lower limbs can help mitigate the effects of aging, preserving structural and cognitive function [25,26,27]. Engaging in individualized exercise programs promotes high levels of motivation, participation, and positive self-perception [28,29]. The participants who became more aware of the risks associated with sedentary behavior recognized the benefits of resistance training for daily life. Different studies highlight how resistance exercise can improve strength levels while preventing or reducing conditions such as sarcopenia and osteoporosis, which are common among sedentary individuals [30,31,32]. Like many studies, this research presents some limitations. The relatively small sample size may limit the generalizability of the findings, and the absence of a control group prevents direct comparisons with a non-intervention group, which could have strengthened the interpretation of the results. Additionally, the reliability of the survey remains limited due to the small sample size and the limited number of items, despite the acceptable internal consistency indicated by Cronbach’s alpha. The absence of a control group was a methodological choice dictated by the nature of this study, given that the main objective was to observe intra-subject changes over time rather than to compare with an untrained group. Similarly, the survey aimed to assess these intra-subject changes, and its validation would benefit from a larger sample to ensure greater reliability and generalizability. Future research should consider expanding the sample and including a control condition to enhance the robustness and applicability of the findings. In conclusion, this study demonstrates that structured strength development programs not only enhance physical abilities but also cultivate a greater awareness of the importance of exercise. These findings underscore the value of incorporating such protocols into both educational and training environments to promote long-term health and well-being [33].

## 5. Conclusions

The overall findings of this study indicate that increased strength is closely linked to a heightened awareness of the benefits of exercise for improving strength levels. This connection suggests that strength training can be effectively utilized for educational purposes by encouraging greater participation in structured exercise programs. This study also highlights the logical relationships between the quantitative and qualitative–quantitative data, adding both scientific and methodological value to the research. By examining the causal link between strength development and the participants’ perceptions and opinions, the findings demonstrate that an active lifestyle is perceived as a key factor in lifelong learning [34]. These results reinforce the idea that engaging in strength training not only enhances physical performance but also fosters a deeper understanding of the long-term benefits of exercise, promoting a more informed and proactive approach to health and well-being.

## Figures and Tables

**Table 1:** Training protocol, Phase 1: Day 1 and Day 2.

Day 1: Warm-Up				
90/90 mobility, supine pelvic movement, leg lower, hip cook lift 1 leg, Cat–Dog, pull apart, ham stretch. Squat pattern, Deadlift pattern, split squat, linear march, linear march variation.				
Light Implement Power				
Exercises	*W1*	*W2*	*W3*	*W4*
NCM Box Jump (Box 25 cm)	3 × 5	3 × 5	3 × 6	3 × 6
HK Chest Pass	3 × 5	3 × 5	3 × 6	3 × 6
Strength				
Exercises	*W1*	*W2*	*W3*	*W4*
Goblet squat (+2.5 kg X week)	3 × 8	3 × 8	3 × 8	3 × 8
Two Dumbell Floor Press	3 × 8	3 × 8	3 × 8	3 × 8
HK Elevated Front Plank	3 × 20″	3 × 25″	3 × 30″	3 × 35″
Single Leg Deadlift	3 × 8	3 × 8	3 × 8	3 × 8
Day 2: Warm-Up				
90/90 mobility, supine pelvic movement, leg lower, hip cook lift 1 leg, Cat–Dog, pull apart, ham stretch. Squat pattern, Deadlift pattern, split squat, linear march, linear march variation.				
Light Implement Power				
Exercises	*W1*	*W2*	*W3*	*W4*
CM lateral bound	3 × 5	3 × 5	3 × 6	3 × 6
Hip-Hinge HK side toss	3 × 5	3 × 5	3 × 6	3 × 6
Strength				
Exercises	*W1*	*W2*	*W3*	*W4*
Trap Bar Deadlift	3 × 8	3 × 8	3 × 8	3 × 8
Bb Pull-Ups	3 × 8	3 × 8	3 × 8	3 × 8
Side Plank	3 × 20″	3 × 25″	3 × 30″	3 × 35″
Split squat	3 × 8	3 × 8	3 × 8	3 × 8
HK Landmine Oh Press				

**Table 2:** Training protocol, Phase 2: Day 1 and Day 2.

Day 1: Warm-Up				
90/90 mobility, supine pelvic movement, leg lower, hip cook lift 1 leg, Cat–Dog, pull apart, ham stretch. Squat pattern, Deadlift pattern, split squat, linear march, linear march variation.				
Light Implement Power				
Exercises	*W1*	*W2*	*W3*	*W4*
CM Box Jump (Box 25 cm)	3 × 5	3 × 5	3 × 6	3 × 6
Hip-Hinge Chest Pass	3 × 5	3 × 5	3 × 6	3 × 6
Strength				
Exercises	*W1*	*W2*	*W3*	*W4*
Goblet squat	3 × 8	3 × 8	3 × 8	3 × 8
Two Dumbell Floor Press	3 × 8	3 × 8	3 × 8	3 × 8
Elevated Front Plank	3 × 20″	3 × 25	3 × 30″	3 × 35″
Day 2: Warm-Up				
90/90 mobility, supine pelvic movement, leg lower, hip cook lift 1 leg, Cat–Dog, pull apart, ham stretch. Squat pattern, Deadlift pattern, split squat, linear march, linear march variation.				
Light Implement Power				
Exercises	*W1*	*W2*	*W3*	*W4*
CM lateral bound	3 × 5	3 × 5	3 × 6	3 × 6
Hip-Hinge HK side toss	3 × 5	3 × 5	3 × 6	3 × 6
Strength				
Exercises	*W1*	*W2*	*W3*	*W4*
BB Pull-Ups	3 × 8	3 × 8	3 × 8	3 × 8
Trap Bar Deadlift	3 × 8	3 × 8	3 × 8	3 × 8
Side Plank	3 × 20″	3 × 25″	3 × 30″	3 × 35″
Split squat	3 × 8	3 × 8	3 × 8	3 × 8

**Table 3:** Perception survey.

Pre-training (responses on a scale from 1 to 5):	Q1. How aware are you of your current muscle strength capabilities? Q2. How much do you think your muscle strength abilities affect your daily life or performance? Q3. How important do you think it is to work on improving your muscle strength skills?
Mid-training (during this study):	Q1. How much do you think your awareness of muscle strength has improved since the beginning of the protocol? Q2. How much do you think the protocol is affecting your daily life or performance right now? Q3. How motivated do you feel to continue the protocol to achieve further improvements?
Post-training:	Q1. How much do you think your awareness of muscle strength has improved since the beginning of the protocol? Q2. How satisfied are you with the way the protocol has affected your daily life or performance? Q3. How important do you think it is to continue monitoring your muscle strength skills to maintain and further improve your performance?

**Table 4:** HGT results.

Athletes (n = 24)	Right Pre	Right Mid	Right Post	Left Pre	Left Mid	Left Post
Mean	31.74	34.01	37.39	31.05	33.83	36.97
SD	8.50	8.30	8.83	8.26	8.13	8.09

**Table 5:** ANOVA results and post hoc analysis for the Hand Grip Test.

Variable	F	*p*-Value	Comparison	*p*-Value
Right	2.65	0.07	N.A.	N.A.
Left	3.16	0.04	pre vs. mid	0.00
			pre vs. post	0.00
			mid vs. post	0.06

Note: N.A., non-applicable.

**Table 6:** Descriptive statistics IMTP results.

Athletes (n = 24)	Mean Force (kg) Pre	Mean Force (kg) Mid	Mean Force (kg) Post
Mean	97.69	105.45	108.06
SD	20.04	20.57	20.26

**Table 7:** Mean perception scores across the three time points.

	Pre Q1	Pre Q2	Pre Q3	Mid Q1	Mid Q2	Mid Q3	Post Q1	Post Q2	Post Q3
Mean	2.41	2.50	2.58	3.37	3.41	3.45	4.33	4.41	4.62
SD	0.50	0.58	0.65	0.57	0.50	0.58	0.56	0.50	0.49

**Table 8:** Friedman test results and post hoc analysis for athlete perceptions.

χ^2^_F_	*p*-Value	Comparison	*p*-Value	d (95% CIs)
8.71	0.01	pre vs. mid	0.00	0.62 (0.45, 0.78)
		pre vs. post	0.00	0.38 (0.20, 0.56)
		mid vs. post	0.01	0.38 (0.20, 0.56)

## Data Availability

All data generated or analyzed during this study are included within the manuscript.
